# The mdx Mutation in the 129/Sv Background Results in a Milder Phenotype: Transcriptome Comparative Analysis Searching for the Protective Factors

**DOI:** 10.1371/journal.pone.0150748

**Published:** 2016-03-08

**Authors:** Priscila Clara Calyjur, Camila de Freitas Almeida, Danielle Ayub-Guerrieri, Antonio Fernando Ribeiro, Stephanie de Alcântara Fernandes, Renata Ishiba, Andre Luis Fernandes dos Santos, Paula Onofre-Oliveira, Mariz Vainzof

**Affiliations:** Laboratory of Muscle Proteins and Comparative Histopathology, Human Genome and Stem-cell Research Center, Biosciences Institute, University of São Paulo, São Paulo, Brazil; Stem Cell Research Institute, BELGIUM

## Abstract

The mdx mouse is a good genetic and molecular murine model for Duchenne Muscular Dystrophy (DMD), a progressive and devastating muscle disease. However, this model is inappropriate for testing new therapies due to its mild phenotype. Here, we transferred the mdx mutation to the 129/Sv strain with the aim to create a more severe model for DMD. Unexpectedly, functional analysis of the first three generations of mdx^129^ showed a progressive amelioration of the phenotype, associated to less connective tissue replacement, and more regeneration than the original mdx^C57BL^. Transcriptome comparative analysis was performed to identify what is protecting this new model from the dystrophic characteristics. The mdx^C57BL^ presents three times more differentially expressed genes (DEGs) than the mdx^129^ (371 and 137 DEGs respectively). However, both models present more overexpressed genes than underexpressed, indicating that the dystrophic and regenerative alterations are associated with the activation rather than repression of genes. As to functional categories, the DEGs of both mdx models showed a predominance of immune system genes. Excluding this category, the mdx^129^ model showed a decreased participation of the endo/exocytic pathway and homeostasis categories, and an increased participation of the extracellular matrix and enzymatic activity categories. *Spp1* gene overexpression was the most significant DEG exclusively expressed in the mdx^129^ strain. This was confirmed through relative mRNA analysis and osteopontin protein quantification. The amount of the 66 kDa band of the protein, representing the post-translational product of the gene, was about 4,8 times higher on western blotting. *Spp1* is a known DMD prognostic biomarker, and our data indicate that its upregulation can benefit phenotype. Modeling the expression of the DEGs involved in the mdx mutation with a benign course should be tested as a possible therapeutic target for the dystrophic process.

## Introduction

Neuromuscular disorders are a heterogeneous group of genetic diseases, causing progressive loss of motor ability. More than 30 genetically defined forms are recognized and in the last decade mutations in several genes have been reported that result in deficiency or loss of function of different important muscle proteins.

Duchenne muscular dystrophy (DMD) is the most common and severe human muscular dystrophy, affecting 1 in 3500 male births. It is caused by mutations in the dystrophin gene which result in the absence of this important sarcolemmal protein and consequent muscle degeneration. The clinical course of DMD is severe and progressive, starting with muscle weakness at the age of five and loss of ambulation around 12 years; without special care, death occurs due to respiratory failure or cardiomyopathy in the late teens [1]. There is no effective cure for patients suffering from this type of dystrophy.

Several animal models manifesting phenotypes observed in neuromuscular diseases have been identified in nature or generated in laboratory. These models generally present physiological alterations observed in human patients and can be used as important tools for pathophysiological studies and therapy testing [2]. The C57BL/10ScSn-*Dmd*^*mdx*^/J, named here as mdx, is the most widely used animal model for DMD, bearing a non-sense point mutation in exon 23 of the dystrophin gene which causes lack of this protein in the skeletal muscle [3]. However, differently from human DMD patients, the mdx presents a mild phenotype, with normal lifespan and reproductive capacity [4]. Therefore, this model is not effective in clinical trials to track possible functional benefits of tested therapies.

Studies in animal models by insertion of a human pathogenic mutation in distinct mouse backgrounds have shown differences in phenotypical manifestation according to the mouse strain. Previously, a group introduced the mgΔ mutation from a mouse model for Marfan syndrome in two different genetic backgrounds: C57BL and 129/Sv [5]. The animals with 129/Sv background presented a more severe and earlier phenotype than those with C57BL background.

Considering that the increased severity of muscle damage is extremely useful in assessing how effective a novel therapy might be at halting human disease and in order to obtain a more reliable animal model for DMD, we decided to transfer the mdx mutation to the 129/Sv background expecting that the resulting animals would present a more severe DMD phenotype.

Unexpectedly, the newly created mdx^129^ model showed a progressive amelioration of the phenotype. Thus, a functional analysis, followed by transcriptome comparative gene expression analysis, histological and protein studies were performed to identify and characterize what is protecting this new model from the dystrophic characteristics. We identified the overexpression of the *Spp1* gene, which codes for the osteopontin (OPN) protein, as the most significant candidate to cause this benefit to the dystrophic phenotype.

## Materials and Methods

### Animals

The 129/Sv male mice were obtained from the ICB USP experimentation housing facility, while C57BL and the mdx females were obtained from the Center for Human Genome and Stem Cell Researches (IB USP) experimentation housing facility. The animals were kept under controlled temperature and light conditions and were fed with pellets and water ad libitum. All experimental procedures were analyzed and approved by the Institute of Biosciences Ethics Commission in the Use of Animals (Permit Number: CEUA/IBUSP 201/2014).

### Transferring the mdx mutation to the 129/Sv phenotype

**Fig 1:**
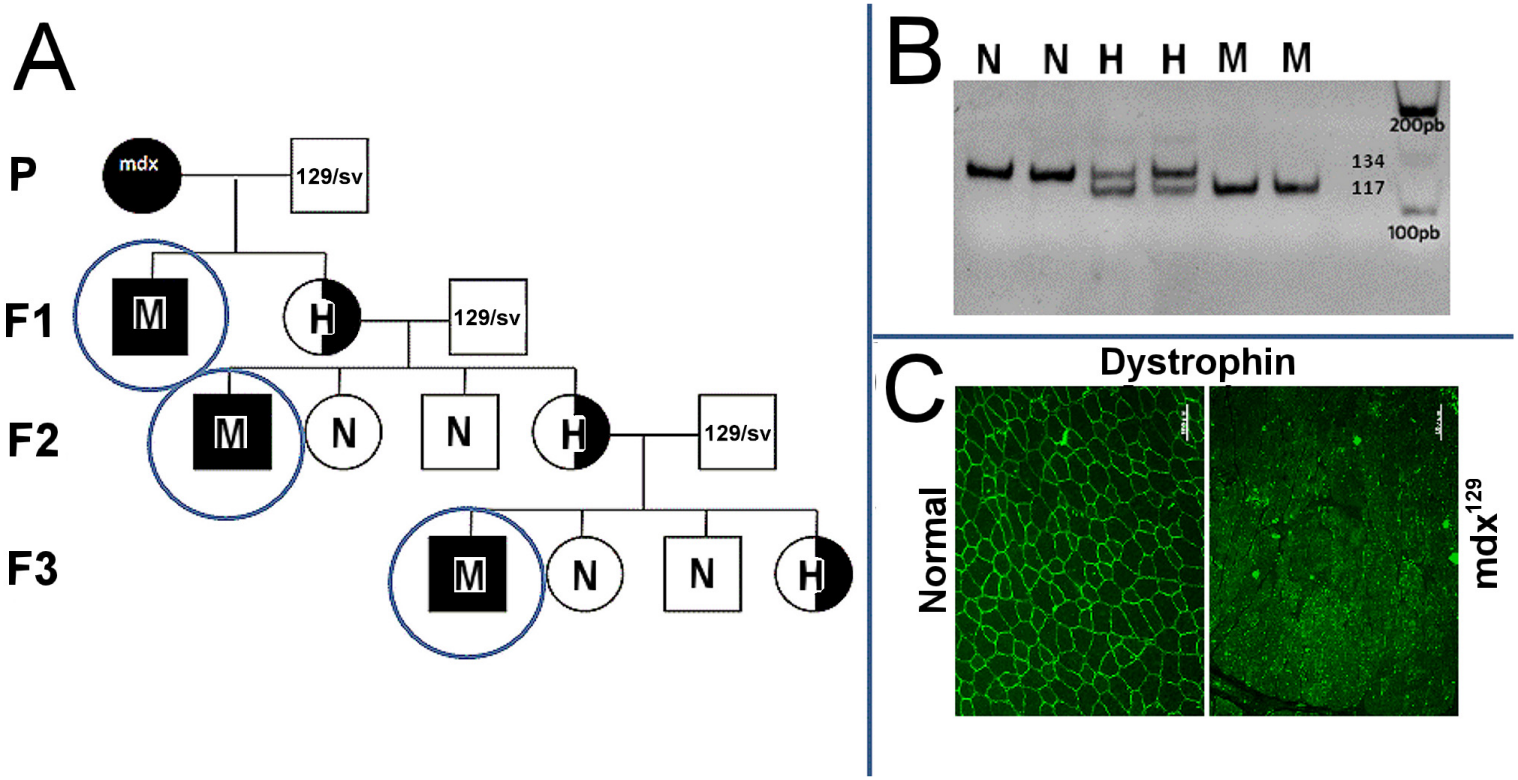
Originating the mdx^129^ mouse. (A) Schematic representation of the cross-breeding. (B) Genotyping for the mdx mutation: acrylamide gel electrophoresis of the PCR competitive reaction showing the presence of the 134 pb band in two wild type normal DNA (N), a 117 pb band in the mdx (M) and both bands in two carrier females (H). (C) Dystrophin immunofluorescence analysis with DYS2 antibody showing the presence of dystrophin in the muscle membrane of normal control, and the absence of dystrophin in the mdx^129^.

The first breeding pairs consisted of 129/Sv males and mdx females. Their offspring (generation mdx^129^ F1) consisted of affected males and carrier females. The carrier females were backcrossed with the 129/Sv males, and their offspring (generation mdx^129^ F2) consisted of wild-type males and females, affected males and carrier females, according to Mendelian proportions (Fig 1A). From this generation on, the litters were genotyped for the mdx mutation to select only the affected males and carrier females (Fig 1B). These carrier females were then backcrossed with the 129/Sv male, generating mdx^129^ F3.

### Genotyping

DNA was extracted from a 0.5cm piece of tail using Proteinase K (Promega, Madison, WI, USA) as described [6]. The genotyping was done by PCR competitive reaction, using specific primers for the exon 23 of the murine dystrophin gene. The product was applied in 10% acrylamide gel where different band patterns can be identified for wild type (134 pb), heterozygous (134 and 117 pb) and affected animals (117 pb) (Fig 1B), according to a previously described protocol [7]. Dystrophin analysis confirmed the deficiency of the protein in F3 males, carrying the pathogenic mutation in the dystrophin gene (Fig 1C).

### Functional evaluations

All mdx^129^ male mice obtained in the three generations were monthly evaluated during the period of six months, using mdx^C57BL^ mice as controls. The number of studied male animals was: F1 = 9, F2 = 13, F3 = 14.

The used tests were previously described [8] and validated in our colonies of neuromuscular disease mice models. In addition, the same researcher performed the analysis in all mice, avoiding intra-personal variability. Briefly:

1. The animals capacity of hanging from a bar by their fore limbs and by all four limbs—the animal is positioned hanging from a 3mm metal bar by its fore limbs or by all four limbs and the time it keeps hanging is counted. We consider 60 seconds to be the maximum time and the test is successively repeated for three times, after which we calculate the average.
2. Fore limbs and hind limbs grip strength—the animal is positioned so that it will grip the grid attached to a dynamometer with its fore limbs or with its hind limbs and then is pulled by the tail until it releases it. This procedure is successively repeated for five times and the mean is calculated.

### Histological and immunohistochemical analyses

For these analyses, three male animals from each group were used, all in the age of six months. After animals were euthanized, muscles of the posterior portion of the leg (mainly encompassing the gastrocnemius muscle) and the diaphragm were dissected, fixed in cork blocks with Tissue-Tek^®^ O.C.T Compound (Sakura Finetek USA, Torrance, CA, USA), cryoprotected with talc and frozen in liquid nitrogen.

For histopathology analysis, the following parameters were used: percentage of centronucleated fibers (evaluated using hematoxilin/eosin (HE) staining), fibrosis (evaluated using the quantification of picrossirius staining) and regenerating fibers (quantified using immunofluorescence staining for mouse monoclonal myosin heavy chain (developmental) antibody (VP-M664, Vector Laboratories, Burlingame, CA, USA), in a double reaction with antibody to muscle laminin (rat monoclonal anti-laminin gamma 1 antibody, ab80580, Abcam, Cambridge, MA, USA). Additionally, a mouse monoclonal anti-dystrophin DYS2 antibody was used to identify this protein (VP-D505, Vector Laboratories, Burlingame, CA, USA). The slides were examined and photographed using a Zeiss AxioImager.Z1 microscope.

For positive developmental myosin fibers quantification, fiber numbers from at least five different fields in the cross-sections from each animal were measured, and the total number of positive fibers was compared to the total number of counted fibers. The number of counted fibers was in a total of 600 to 2000 fibers per animal. The area of fibrotic tissue using picrossirius-stained sections was relatively measured using ImageJ software (http://rsb.info.nih.gov/ij/) and using area of control mouse as 1.

### Western blotting analysis

Total proteins were extracted from the gastrocnemius muscle and separated by 13% SDS-PAGE polyacrylamide gel electrophoresis and were transferred onto a nitrocellulose membrane (GE Healthcare Biosciences, Pittsburgh, PA, USA) for 60 min at 0.35A at 4°C. Membranes were then pre-stained in 0.2% Ponceau S, to ensure protein transfer and equal protein loading of the lanes. Membranes were blocked with 5% non fat milk in PBS, 0.1% Tween 20 (TBS-T) for 1 h and probed using the primary antibodies against OPN sc-21742 (Santa Cruz Biotechnology, Inc., Dallas, TX, USA). After an overnight period of incubation, membranes were washed three times with TBS-T for 10 min. The blots were then immunostained with Pierce^®^ Anti-mouse IgG (HRP) Polyclonal antibody (Thermo Fisher Scientific, Waltham, MA, USA) and posterior detection of protein was done using Novex^®^ ECL Chemiluminescent Substrate Reagent Kit (Invitrogen, Waltham, MA, USA).

Quantitative analysis of mouse OPN present in total protein extracted was performed using the ImageJ software (http://rsb.info.nih.gov/ij/), with myosin at the Ponceau staining as a protein loading control. The value of each animal was normalized to the normal control within the same blot.

### RNA extraction and purification

For RNA extraction, frozen gastrocnemius muscle of three animals from each strain was used, at the age of six months. Frozen muscles were finely powdered using a mortar and total RNA was extracted using RNeasy Microarray Tissue Mini Kit (Qiagen, Valencia, CA, USA) according to manufacturer instructions. RNA contamination by DNA was verified in 1% agarose gel.

### Hybridization and Microarray data analysis

Samples for hybridization were prepared using Ambion^®^ WT Expression and GeneChip^®^ WT Terminal Labeling (Life Technologies, Waltham, MA, USA) and hybridized in GeneChip^®^ Mouse Gene 1.0 ST Array (Affymetrix, Santa Clara, CA, USA) chips, all according to instructions provided by the manufacturer. Pre-analysis and data normalization were performed in the Expression Console^™^ (Affymetrix, Santa Clara, CA, USA) software using the RMA (Robust Multi-array Average) algorithm. Normalized data were uploaded to the MeV software where differentially expressed genes (DEGs) were determined by the SAM algorithm. To study functional networks among the identified DEGs, we used the IPA software (Ingenuity^®^ Systems, www.ingenuity.com). Gene ontology function enrichment analysis was performed with the DAVID tool (Database for Annotation, Visualization and Integrate Discovery http://david.abcc.ncifcrf.gov/). Raw data has been deposited at Gene Expression Omnibus (GEO) database (accession number GSE77126).

### qPCR analysis

RNA was quantified and normalized for cDNA synthesis using oligo dT and random primers, and MMLV enzyme (Invitrogen, Waltham, MA, USA), in a quick 3-step protocol of incubation in 65°C or 37°C. For qPCR, we used the protocol previously described [9]. Samples of cDNA of each animal were applied in triplicate in 96 wells plates. At each sample was added the pair of primers of the gene of interest and Sybr Green Master Mix (Applied Biosystems, Carlsbad, CA, USA) in a total volume of 25μL. Each plate was run in the 7500 Fast Applied Biosystems thermocycler for real-time PCR.

The relative expression of the following genes was measured:

- Pathway of muscle regeneration: *MyoD*, *Myf5* and *Myogenin* (*Myog*);
- Validation of microarray data: *Spp1* and *Col5a2*.

The used primers are described in Table 1: we used previously described primers for *Spp1* [10] and the others were selected from a real-time primer database with validation results (Primerbank). The chosen gene for use as an endogenous control was *Gapdh*.

**Table 1:** Primers used in relative quantification of gene expression.

Gene	Forward	Reverse
***MyoD***	TACAGTGGCGACTCAGATGC	TAGTAGGCGGTGTCGTAGCC
***Myf5***	CTGTCTGGTCCCGAAAGAAC	GACGTGATCCGATCCACAATG
***Myogenin***	CAGTACATTGAGCGC CTACAG	GGACCGAACTCCAGTGCAT
***Spp1***	AGCAAGAAACTCTTCCAAGCAA	GTGAGATTCGTCAGATTCATCCG
***Col5a2***	TTGGAAACCTTCTCCATGTCAGA	TCCCCAGTGGGTGTTATAGGA
***Gapdh***	AGGTCGGTGTGAACGGATTTG	TGTAGACCATGTAGTTGAGGTCA

As the analysis uses one unique calibrator control, it was necessary to select one strain, for this purpose. We decided to use the wild type mouse 129/Sv sample with the nearest expression of the most genes in all of them.

### Statistical Analysis

Functional evaluations were statistically analyzed using Mann-Whitney test. For the other parameters, due to the small size of the samples, Mann-Whitney and Kruskal Wallis corrected with Dunn-Bonferroni non parametric tests were performed, but with no statistically significant differences detected. All calculations were performed using the Minitab 17 software.

## Results

### Evidences from functional evaluations

**Fig 2:**
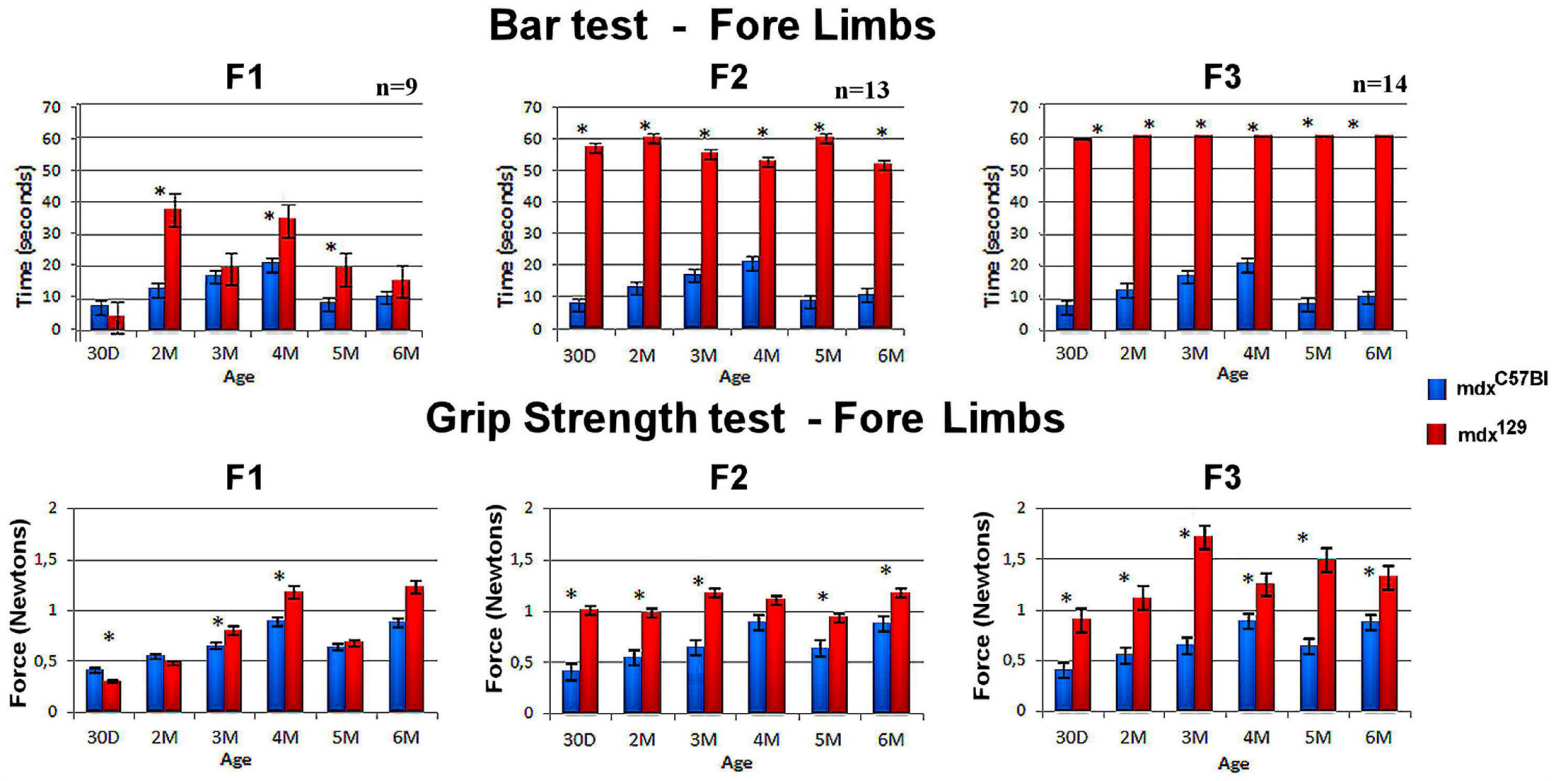
Graphical representation of comparative functional test in the mdx^C57Bl^ as compared to the three mdx^129^ generations. Data of the fore limbs in the bar test and grip strength test are shown with the median expression and standard error range for each age/strain. The number of tested animals in each generation is also presented. * p<0,05.

As shown in Fig 2, in the first generation of mdx^129^ (F1) forelimbs bar test, the animals already demonstrated more resistance than mdx^C57BL^ animals, yet, the results were significant merely in the ages two, four and five months. In the grip strength test, F1 animals were stronger than mdx^C57BL^ mice, but the results were only significant at 30 days, three and four months. The second mdx^129^ generation (F2) showed improved performance in all tests compared to mdx^C57BL^. In both bar tests, F2 mice hang on the bar for the whole time of the test (60 seconds), and the values were statistically significant at all ages. The strength was also increased in this group. Noticing the results from the third mdx^129^ generation (F3), we could confirm the progressive increase both in resistance and strength of mdx^129^ animals. The F3 mice hang on the bar for 60 seconds with no sign of fatigue, and grip strength results were better than in generations F1 and F2. The same results were observed in the test applied for the four limbs resistance and hind limb strength.

Therefore, the observed results were the opposite of what was previously described [5] and which we were expecting. Considering the amelioration of the mdx phenotype in the 129/Sv background, we hypothesized that factors in this strain could act protecting the mdx^129^ from the dystrophic effect. Consequently, the study was directed to identify these factors as possible positive modifiers of the dystrophic phenotype.

### Muscle Analysis

#### Evidence from histological analysis

**Fig 3:**
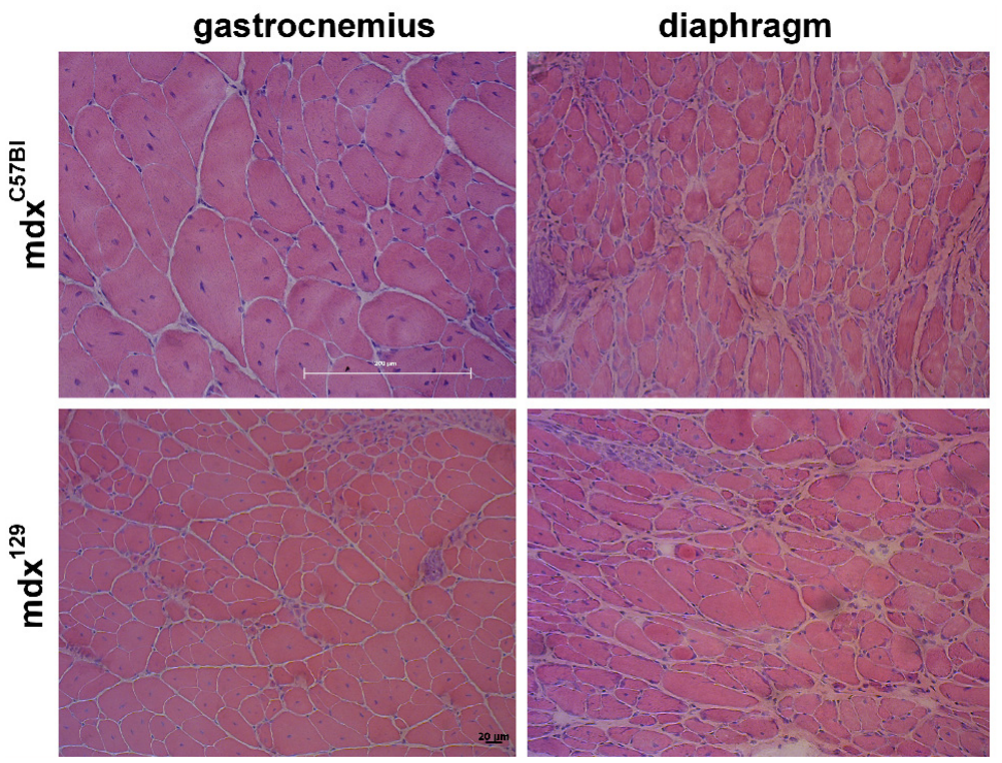
Comparative histological analysis. HE staining of gastrocnemius and diaphragm muscles, in mdx^C57BL^ and mdx^129^ mice in the age of 6 months.

Histological analysis using HE stain showed that the degenerative/regenerative processes were similar in all mdx^C57BL^ mice as compared to the three generations of mdx^129^ mice, both in gastrocnemius and diaphragm muscles (Fig 3). In all strains, degenerated fibers, regenerating fibers and connective tissue infiltration were identified, with almost 100% of centrally nucleated fibers.

#### Evidence of reduced degeneration

**Fig 4:**
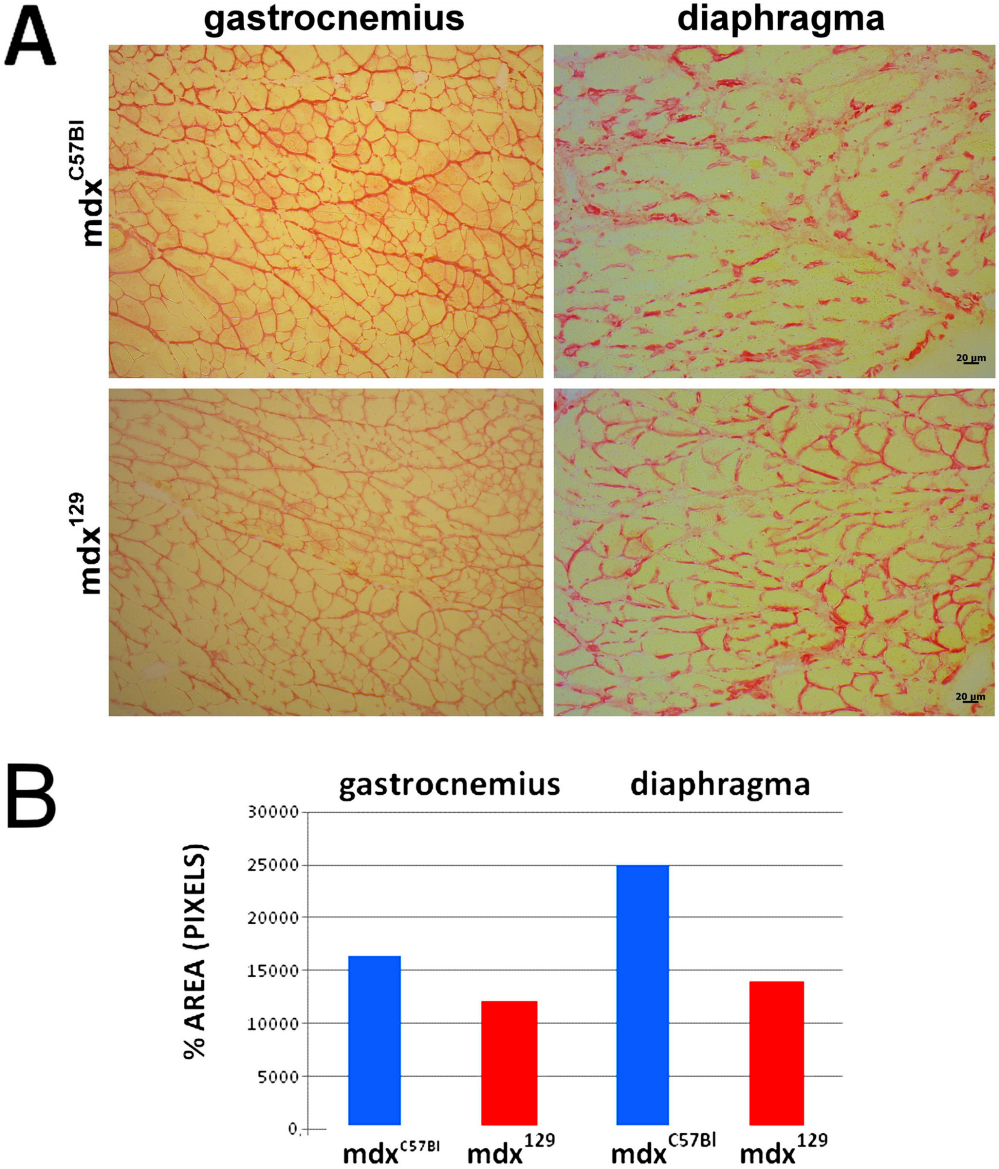
Quantification of connective tissue. (A) Representation of picrossirius staining of gastrocnemius and diaphragm muscles from mdx^C57BL^ and mdx^129^ models. (B) Graphic representing the quantitative comparison between the two mdx strains.

The quantification of connective tissue replacement, measured by picrossirius staining, demonstrated a reduction of the amount of endomysial and perimysial connective tissues in mdx^129^ in relation to mdx^C57BL^, both in gastrocnemius (27%) as well as in the diaphragm (44%) muscles (Fig 4).

#### Evidence of regeneration

**Fig 5:**
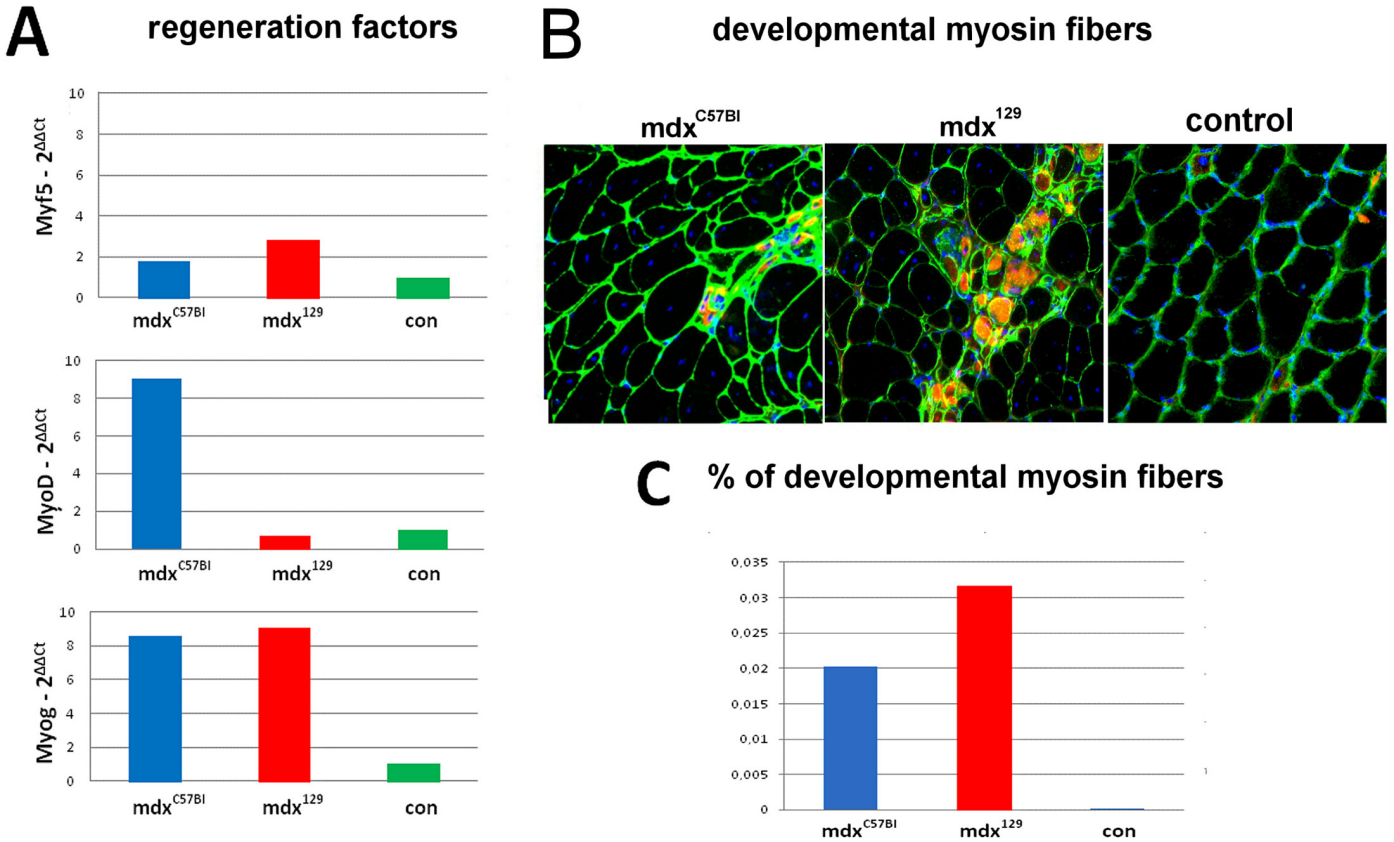
Quantification of the regeneration in the two mdx models. (A) Results of the qPCR expression of genes involved with the regeneration process (*Myf5*, *MyoD* and *Myog*). (B) Representation of the comparative immunohistochemical analysis of gastrocnemius for developmental myosin (red) in double reaction with laminin (green), showing the proportion of positive fibers in mdx^C57BL^ and mdx^129^ mice in the age of 6 months. (C) Graphic representing the quantitative comparison between the two groups of mdx with normal control (con).

The regeneration findings obtained for gastrocnemius samples of normal (129/Sv) and the two mdx models are shown in Fig 5. The average relative expression for regenerative genes (*Myf5*, *MyoD* and *Myog*) was obtained in qPCR analysis (Fig 5A). As to myogenic factors related to early regeneration, *Myf5* expression was higher in mdx^129^ mice versus control, while *MyoD* expression was higher in mdx^C57BL^. However, for the late stage of myogenic differentiation, *Myog* expression presented higher expression in the two mdx models when compared to control. Meanwhile, immunohistochemical analysis for developmental myosin in gastrocnemius muscle of the two affected mice models showed that mdx^129^ presented an increase of about 50% in the number of these fibers in relation to the mdx^C57BL^, suggesting a more active regeneration in mdx^129^ (Fig 5B and 5C).

#### Evidence from transcriptome analysis

We used expression microarray to investigate mdx^129^ transcriptome comparing it to mdx^C57BL^ and each of these models to their same background wild type.

#### C57BL and 129/Sv background comparison

First, we compared both wild types to verify the difference between them. In the comparison of C57BL/ x 129/Sv we found 44 differentially expressed genes (DEGs), and 13 DEGs among the muscle expressed genes. The majority of these DEGs were downregulated in 129/Sv in comparison to C57BL (Table 2).

**Table 2:** Comparative transcriptome analysis.

Tested groups	Total DEGs		Skeletal muscle filter DEGs	
**C57BL x 129/Sv**	44	↑ 11	13	↑ 1
		↓ 33		↓ 12
**C57BL x mdx**^**C57BL**^	371	↑ 320	135	↑ 107
		↓ 51		↓ 28
**129/Sv x mdx**^**129**^ **F3**	137	↑ 130	59	↑ 58
		↓ 7		↓ 1
**mdx**^**C57BL**^ **x mdx**^**129**^ **F3**	36	↑ 19	14	↑ 7
		↓17		↓ 7
**mdx**^**129**^ **F1 x mdx**^**129**^ **F2**	3	↓ 3	1	↓ 1
**mdx**^**129**^ **F1 x mdx**^**129**^ **F3**	5	↑ 1	2	↑ 1
		↓ 4		↓ 1
**mdx**^**129**^ **F2 x mdx**^**129**^ **F3**	0		0	

These data showed the number of identified differentially expressed genes (DEGs), and the proportion of up and downregulated genes in each comparison. The analysis was done in total transcripts and also using a filter selecting genes expressed in the muscle.

#### Comparing both mdx models

Comparing C57BL and mdx^C57Bl^, we found 371 DEGs: 320 upregulated and 51 downregulated in mdx^C57BL^. When comparing 129/Sv with mdx^129^ F3, we found 137 DEGs: 130 upregulated and 7 downregulated in mdx^129^ F3 mice. In both backgrounds, the number of upregulated genes was higher than the downregulated ones (Table 2).

The DEGs were classified according to their functional categories and we observed that the immune system category was the most significant, gathering 60% of the genes in C57BL x mdx^C57BL^ and 80% of the genes in 129/Sv x mdx^129^.

**Fig 6:**
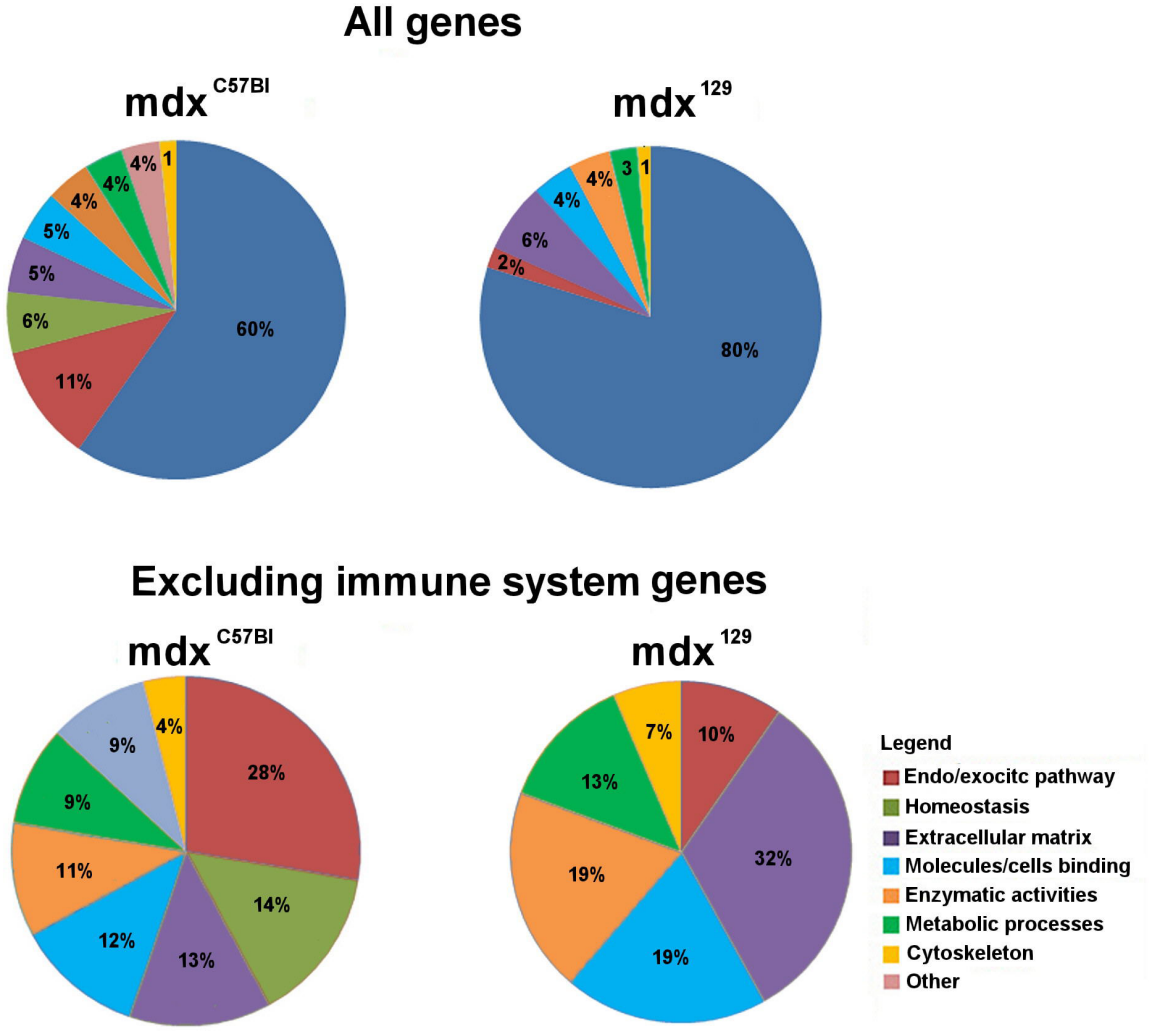
Graphic representation of the distribution in functional categories. (A) Total identified DEGs in the comparisons C57BL x mdx^C57BL^ and 129/Sv x mdx^129^ F3. (B) Categories excluding the immune system.

Since this category is so predominant, we decided to remove it in order to verify the significance of the participation of other categories in the two mdx models (Fig 6).

Both models showed corresponding categories, except homeostasis, which only appear in C57BL x mdx^C57BL^. Otherwise, the proportion in which these categories appear is different. Genes related to endo/exocytic pathways demonstrated a decreased expression in 129/Sv x mdx^129^, while an increased expression was found in genes of other categories, such as extracellular matrix, binding to molecules/cells and enzymatic activity.

#### Comparing both mdx models for genes expressed in muscle

**Fig 7:**
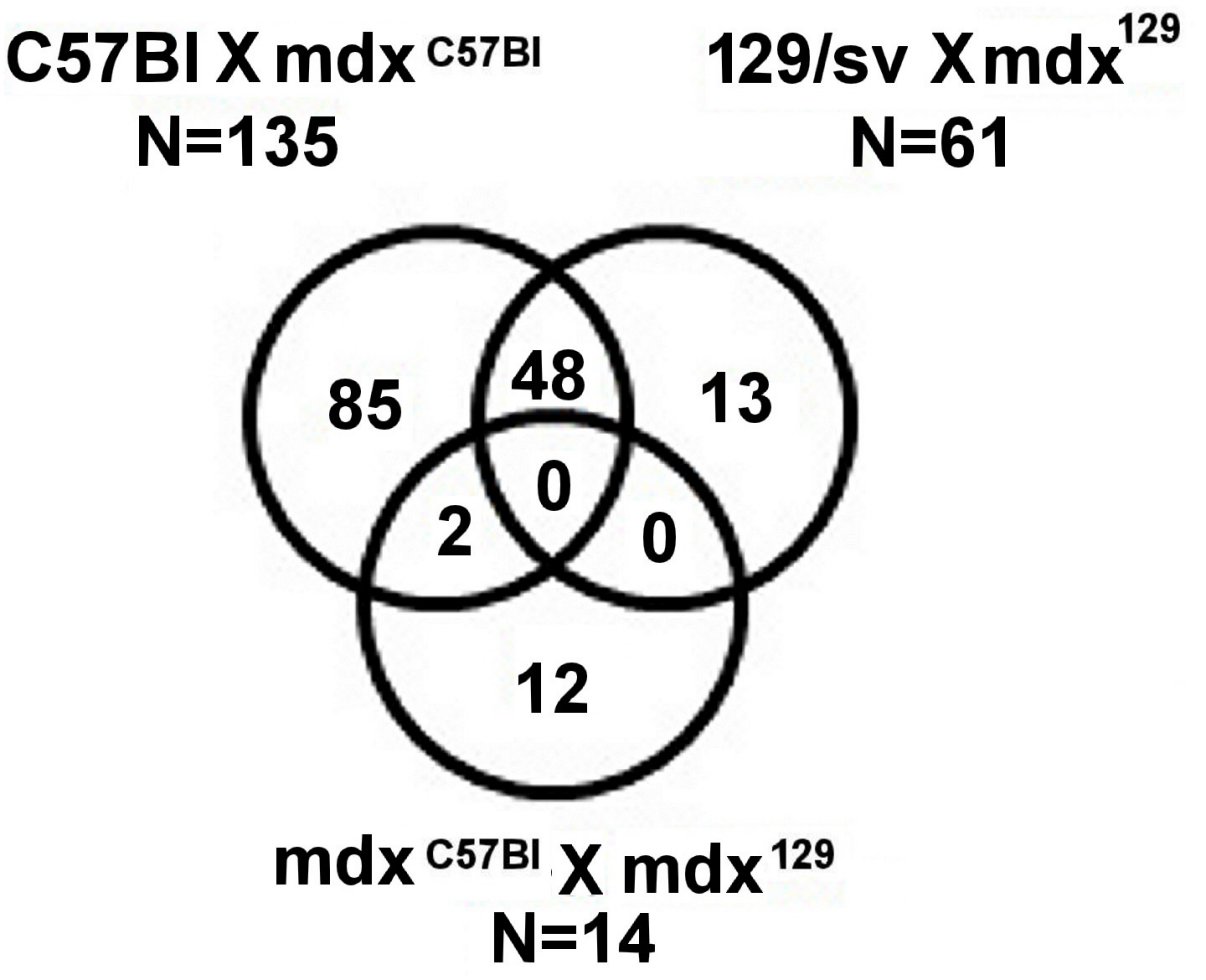
Venn diagram showing genes in common and exclusive DEGs in each of compared lists.

To better visualize the changes in mdx^129^ expression profile, we filtered the lists C57BL x mdx^C57BL^, 129/Sv x mdx^129^ and mdx^C57BL^ x mdx^129^ for genes that, according to the literature, act in skeletal muscle. We found 85 DEGs that were exclusive to C57BL x mdx^C57BL^, 13 DEGs unique to 129/Sv x mdx^129^ and 12 DEGs exclusive to mdx^C57BL^ x mdx^129^ (Fig 7).

Of the 13 genes exclusively expressed in 129/Sv x mdx^129^ (Table 3), all of them presented an upregulated expression, and two important DEGs were identified: *Spp1* and *Ilrn*. Comparing mdx^C57BL^ x mdx^129^ we found 12 exclusive genes (Table 3), where *Klk3* showed the most upregulated expression and *Mup1*, the most downregulated expression. qPCR validated the results observed for *Col5a2* and *Spp1*.

**Table 3:** List of DEGs exclusive of the mdx^129^.

	Comparing 129/Sv x mdx^129^			Comparing mdx^C57BL^ x mdx^129^	
Fold Change	Gene	Entrez Gene Name	Fold Change	Gene	Entrez Gene Name
**1,417**	*Col5a2*	collagen, type V, alpha 2	**-3,621**	*Mup1* (includes others)	major urinary protein 1
**1,509**	*Maged2*	melanoma antigen family D, 2	**-2,461**	*Clec4m*	C-type lectin domain family 4, member M
**1,529**	*Thbs4*	thrombospondin 4	**-1,36**	*Hla-a*	major histocompatibility complex, class I, A
**1,591**	*Dcstamp*	dendrocyte expressed seven transmembrane protein	**-1,28**	*Dbp*	D site of albumin promoter (albumin D-box) binding protein
**1,634**	*Hist2h3a*	histone cluster 2, H3a	**-1,207**	*Nxpe4*	neurexophilin and PC-esterase domain family, member 4
**1,726**	*Il2rg*	interleukin 2 receptor, gamma	**-1,154**	*Hspa8*	heat shock 70kDa protein 8
**1,783**	*P4ha3*	prolyl 4-hydroxylase, alpha polypeptide III	**-1,099**	*5330426p16rik*	RIKEN cDNA 5330426P16 gene
**1,896**	*Top2a*	topoisomerase (DNA) II alpha 170kDa	**1,132**	*Rhobtb3*	Rho-related BTB domain containing 3
**1,974**	*Il1rn*	interleukin 1 receptor antagonista	**1,65**	*Ppp1r3c*	protein phosphatase 1, regulatory subunit 3C
**2,228**	*Tnc*	tenascin C	**1,803**	*Esco1*	establishment of sister chromatid cohesion N-acetyltransferase 1
**2,423**	*Plek*	Pleckstrin	**1,837**	*Ifi202b*	interferon activated gene 202 B
**2,812**	*Cd52*	CD52 antigen	**3,547**	*Klk3*	kallikrein-related peptidase 3
**4,588**	*Spp1*	secreted phosphoprotein 1			

**Fig 8:**
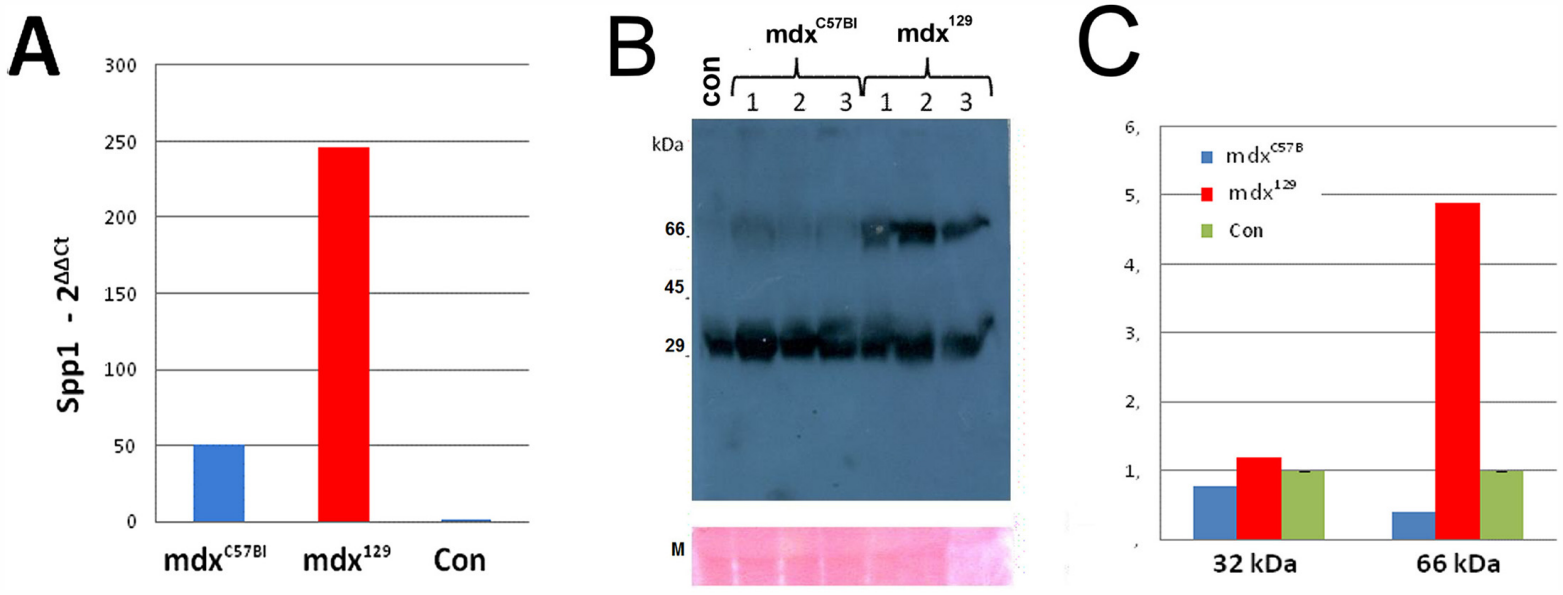
Quantification of *Spp1* transcript and OPN protein expression. (A) Fold changes in qPCR relative expression levels of osteopontin mRNA, as compared to the control group (129/Sv). (B) Western blotting analysis for OPN protein showing both the full length protein (66 kDa band) and one fragment of 32 kDa (cleaved product) in mdx^C57BL^ and mdx^129^ as compared to normal control 129/Sv (con); M—myosin band. (C) Western blotting quantification showing the mean of each group for the band of 32 kDa and 66 kDa. An increase of 4,8 times of the 66 kDA band is observed in the mdx^129^ group using myosin band as a protein loading control.

The most highly induced transcript in the mdx^129^ data set was *Spp1*, which is a strong indicator of muscle injury [11] and an important gene for the dystrophic process. Based on this, *Spp1* was also studied through qPCR for mRNA relative quantification and protein quantification through western blotting (Fig 8). An elevation of ~200X in mRNA expression was observed in mdx^129^ as compared to 129/Sv (Fig 8A). At the protein level, the used OPN antibody recognized both the full-length protein (66 kDa band) and one fragment of 32 kDa corresponding to a cleaved product. Those findings revealed the presence of the same concentration of cleaved product in all mdx models, but a stronger concentration of the full protein (4,8 times higher) in mdx^129^ (Fig 8B and 8C), showing a direct correlation with transcript findings.

## Discussion

Studies in animal models are essential for testing therapies, mainly in diseases still with no effective cure, such as Duchenne Muscular Dystrophy (DMD). Considering that the genetic background can significantly affect phenotype in mouse models of human diseases, and that the most common animal model for DMD, the mdx mouse in the C57BL background, shows a very mild phenotype, we aimed to create a mdx model with a different genetic background, expecting that the resulting animals would present a more severe DMD phenotype. Surprisingly, the opposite results were obtained, with mdx^129^ mice presenting a significantly amelioration of phenotype in all functional tests, in successive generations, when compared to mdx^C57BL^. We could attribute this crescent improvement of the phenotype to the increasing proportion of the 129/Sv background.

We speculated if in the same background of the mouse, primary mutations in different genes could affect phenotype in a different way. Mainly because the mgΔ mutation for Marfan syndrome resulted in a more severe phenotype in the 129/Sv than in the C57BL background [5], while with the mdx mutation the result was inverse: a better phenotype in the 129/Sv than in the C57BL background.

Histological analysis was not informative to differentiate the two mdx strains, since both mdx models presented a similar histopathological pattern in hind limbs as well as in diaphragm muscles. However, a lower proportion of connective tissue and an intense regeneration were observed in mdx^129^ strain, as revealed by higher expression of muscle myogenesis factors and the presence of a higher number of developmental myosin positive fibers in this model.

To identify the protective factors that could be involved, we used an expression microarray analysis to investigate mdx^129^ transcriptome comparing it to mdx^C57BL^, in six-month old animals, when the disease is fully established.

A first remarkable observation was the similarity of C57BL versus 129/Sv wild backgrounds, presenting a small number of DEGs among the mapped genes. Besides, we did not find among the DEGs expressed in the muscle anything which could be, somehow, associated to a directly related muscular function that could eventually provide protection against dystrophy.

As to the comparison between the complete transcriptome of the two mdx strains, we verified that DEGs number is about three times higher in mdx^C57BL^ than in mdx^129^ when compared to wild type from the same background animals. Nevertheless, in both backgrounds the dystrophin gene mutation causes more upregulation than downregulation of genes expression. These results suggest that, in the new background, the dystrophin mutation clearly induced fewer mechanisms of action that could be responsible for the installation and maintenance of the dystrophic process.

The comparison of the three generations of mdx^129^ exhibited a small number of DEGs, and we could therefore conclude that in the expression level, the background effect is present since the first generation, and it does not increase with 129/Sv background (Table 2).

In both models, the participation of genes involved in immune system was clearly predominant, which was expected, once chronic inflammation is a dystrophic muscle characteristic [12]. Nevertheless, when excluding this category, some differences were noted between the two deficient dystrophin models, including a reduction in endo/exocytic pathway but an increase in the participation of the extracellular matrix, binding to molecules/cells and enzymatic activity pathways in mdx^129^. Vesicle trafficking is a necessary process for membrane repair [13] and several studies have shown its importance in the dystrophic phenotype [14, 15]. Our findings suggest that the decreased participation of vesicle-related genes in mdx^129^ animals is due to a more stable membrane, which can present, consequently, better fiber conservation and functional performance. The membrane stability can also explain why the homeostasis category disappeared, since repair mechanism is calcium dependent [16]. Among the functional categories that increased in mdx^129^, the most remarkable was the extracellular matrix. As seen on histological slides, regeneration is present in these animals up to six months; consequently, matrix remodeling is probably occurring, which can explain the increased participation of genes related to this process [17].

When the analysis was focused on genes expressed in muscle, we identified DEGs that were exclusive for some of the compared strains. Among the exclusive DEGs in the comparison of mdx^C57BL^ x mdx^129^ the more upregulated gene was *Klk3* and the more downregulated was *Mup1*. *Klk3* codes for PSA (prostate-specific antigen) which is a prostate cancer biomarker [18]. That being, we could not find a correlation between this gene and mdx^129^ phenotype. *Mup1* is part of a gene family involved in chemical communication among animals (mice and rats) [19] and few studies were conducted about its metabolic functions. A group [20] observed that the skeletal muscle was a major target for *Mup1*. In their study with obese mice, which present lower levels of the protein, partial level corrections of the protein alleviated insulin resistance and glucose intolerance, ameliorating skeletal muscle mitochondrial function. In our study, mdx^129^ presented a significant decrease of *Mup1* expression than mdx^C57BL^, suggesting a beneficial effect of this lower expression in their phenotype. More studies will be needed to explain *Klk3* and *Mup1* roles in muscle function.

Of the 13 genes exclusively expressed in 129/Sv x mdx^129^, two deserve to be highlighted: the upregulated *Spp1* and *Il1rn*. *Il1rn* gene codes for IL-1Ra, an interleukin-1 antagonist. Considering interleukins pro-fibrotic and pro-inflammatory effects, the increased expression of its antagonist suggests that mdx^129^ animals could be more protected from the inflammatory process generated by these molecules.

*Spp1* gene codes for osteopontin (OPN), which can be found as an extracellular matrix component and a soluble molecule with cytokine properties [21]. OPN is a multifunctional molecule that is involved in both physiological and pathological processes, including tissue repair, inflammation and fibrosis [22, 23, 24, 25, 26, 27, 28, 29, 30], and its effects in muscular dystrophy are not yet clear. OPN alterations have been described as a dystrophic and injured muscle component [11, 12, 31].

Our study showed a higher expression of *Spp1* gene in mdx^129^ model in the transcriptomic analysis, and this data was confirmed through mRNA relative quantification and protein quantification through western blotting. OPN overexpression could, therefore, be considered a good candidate to explain the better phenotype of this model. A 100-fold increase in the expression of osteopontin mRNA in regenerating muscle of mice with muscle induced damage, lead Hoffman et al. [32] to suggest that OPN is involved in the inflammatory, degenerative and regenerative events that occur in early skeletal muscle regeneration. Our results strongly support this hypothesis, once we found differences of expression of early regenerative genes (*Myod* and *Myf5*) between the mdx^129^ mice and the other mice strains, suggesting that OPN contributes with muscle regeneration. Additionally, according to the model proposed by Pagel et al. in 2014 [33], at later stages in muscle regeneration, osteopontin may be increasingly incorporated into the remodeled extracellular matrix and support their terminal differentiation into new muscle fibers [34]. As OPN is immobilized by incorporation into the extracellular matrix in terminal differentiation, the fusion of myoblasts is favored, thereby promoting the formation of myotubes. In our study, among the functional categories that increased in mdx^129^, the most remarkable was the extracellular matrix. Also, differences in *Myog* expression and the higher number of positive developmental myosin fibers in the mdx^129^ model support a stimulus for a better terminal differentiation into new muscle fibers in this model. Hence, it is quite likely that the enhancement in OPN expression might play a part in improving the dystrophic pathology in mdx^129^ mice stimulating late regeneration. These findings points to a potential value of OPN as a skeletal muscle disease progression biomarker.

Phenotypic variability due to genetic modifiers that regulate disease process acting in the regeneration process has been demonstrated recently in the DBA/2J background. The mdx mutation in this background confers a more severe muscular dystrophy phenotype than the original strain, demonstrating the presence of genetic modifier loci in the DBA/2J background [35]. Interestingly, self-renewal efficiency of satellite cells in this background is lower than that of C57BL strain [36], demonstrating a direct effect of modifier genes in the capacity of muscle regeneration, an important factor in muscular dystrophy prognosis.

At the molecular level, *Spp1* gene is expressed by a single copy gene as a 34 kDa nascent OPN protein composed of 300 amino acids residues. In mammalian cells, the final size of OPN can ranges from 44 to 75 kDA after the post-translational modifications (glycosylation, sulphatation and phosphorylation), which under certain circumstances influence its function [37, 38]. Western blotting results in our mdx models showed that the full length form of OPN (66 kDa) was present in a higher quantity in mdx^129^ mice suggesting also a more effective post-translational process in this model. On the other hand, OPN is also a substrate for some matrix metalloproteinases (MMPs) [39] and the cleavage by these MMPs occurs at a limited number of sites. It has been reported that OPN proteolytic fragmentation is a biological process having physiological importance [40] and the fragments possess greater activity than the full-length forms [41]. The small ~32 kDa fragment observed in all animals in the same intensity could reflect this proteolytic fragment, and in this case, it would be similar in both models. The larger band would be a repository for this protein in the muscle.

To date, studies on the role of osteopontin in skeletal muscle pose almost as many questions as they answer. In dystrophic mouse muscles chronic overexpression of osteopontin appears to be related to decreased muscle strength and fibrosis in mice [33, 42], whereas in muscles from patients with Duchenne muscular dystrophy a SNP associated with osteopontin overexpression *in vitro* has been found to be a significant positive modifier of the disease [43]. Therefore, one possible explanation for the apparently contradictory observations is that osteopontin plays many roles, some of them antagonistic to others in injured muscle, and that post-translational modification, processing, timing, as well as absolute level of osteopontin expression influence the role that it plays.

The results observed in our more benign mdx^129^ model, expressing higher levels of *Spp1* than the mdx^C57BL^, are suggestive of a positive role of this gene as a prognostic biomarker in humans, since it is more expressed in the mildly affected model.

In conclusion, modeling the expression of these differentially expressed genes involved in the benign course of the mdx mutation, in particular *Spp1* gene, should be tested as possible therapeutic targets for the dystrophic process.

## References

[1] Dubowitz V, Sewry CA, Oldfors A. Muscle biopsy: a practical approach. 4rd ed London, UK: Elsevier Health Sciences; 2013.

[2] Vainzof M, Ayub-Guerrieri D, Onofre PC, Martins PC, Lopes VF, Zilberztajn D, et al Animal models for genetic neuromuscular diseases. J Mol Neurosci. 2008; 34: 241–248. 10.1007/s12031-007-9023-9 18202836

[3] Sicinski P, Geng Y, Ryder-Cook AS, Barnard EA, Darlison MG, Barnard PJ. The molecular basis of muscular dystrophy in the mdx mouse: a point mutation. Science. 1989; 244: 1578–1580. 2662404 10.1126/science.2662404

[4] Bulfield G, Siller WG, Wight PA, Moore KJ. X chromosome-linked muscular dystrophy (mdx) in the mouse. Proc Natl Acad Sci USA. 1984; 81: 1189–1192. 6583703 10.1073/pnas.81.4.1189 PMC344791

[5] Lima BL, Santos EJ, Fernandes GR, Merkel C, Mello MR, Gomes JP, et al A new mouse model for marfan syndrome presents phenotypic variability associated with the genetic background and overall levels of Fbn1 expression. PLoS One. 2010; 5(11): e14136 10.1371/journal.pone.0014136 21152435 PMC2994728

[6] Zangala T. Isolation of genomic DNA from mouse tails. J Vis Exp. 2007; 6: 246 10.3791/246 18997894 PMC2557115

[7] Shin JH, Hakim CH, Zhang K, Duan D. Genotyping mdx, mdx3cv, and mdx4cv mice by primer competition polymerase chain reaction. Muscle Nerve. 2011; 43: 283–286. 10.1002/mus.21873 21254096 PMC3051167

[8] Chiavegatto S, Sun J, Nelson RJ, Schnaar RL. A functional role for complex gangliosides: motor deficits in GM2/GD2 synthase knockout mice. Exp Neurol. 2000; 166: 227–234. 11085888 10.1006/exnr.2000.7504

[9] Gosselin LE, Williams JE, Brazeau D, Koury S, Martinez DA. Localization and early time course of TGF-beta 1 mRNA expression in dystrophic muscle. Muscle and Nerve. 2004; 30(5): 645–53.6 15389721 10.1002/mus.20150

[10] Szulzewsky F, Pelz A, Feng X, Synowitz M, Markovic D, Langmann T, et al Glioma associated microglia/macrophages display an expression profile different from M1 and M2 polarization and highly express Gpnmb and Spp1. PLoS One. 2015; 10(2): e0116644 10.1371/journal.pone.0116644 25658639 PMC4320099

[11] Hirata A, Masuda S, Tamura T, Kai K, Ojima K, Fukase A, et al Expression profiling of cytokines and related genes in regenerating skeletal muscle after cardiotoxin injection: a role for osteopontin. Am J Pathol, 2003; 163: 203–215. 12819025 10.1016/S0002-9440(10)63644-9 PMC1868192

[12] Porter JD, Khanna S, Kaminski HJ, Rao JS, Merriam AP, Richmonds CR, et al A chronic inflammatory response dominates the skeletal muscle molecular signature in dystrophin-deficient mdx mice. Hum Mol Genet. 2002; 11: 263–272. 11823445 10.1093/hmg/11.3.263

[13] Han R. Muscle membrane repair and inflammatory attack in dysferlinopathy. Skelet Muscle. 2011; 1: 10 10.1186/2044-5040-1-10 21798087 PMC3156633

[14] He B, Tang RH, Weisleder N, Xiao B, Yuan Z, Cai C, et al Enhancing muscle membrane repair by gene delivery of MG53 ameliorates muscular dystrophy and heart failure in d-Sarcoglycan-deficient hamsters. Mol Ther. 2012; 20: 727–735. 10.1038/mt.2012.5 22314291 PMC3321592

[15] Swaggart KA, Demonbreun AR, Vo AH, Swanson KE, Kim EY, Cai C, et al Annexin A6 modifies muscular dystrophy by mediating sarcolemmal repair. Proc Natl Acad Sci USA. 2014; 111: 6004–6009. 10.1073/pnas.1324242111 24717843 PMC4000833

[16] Reddy A, Caler EV, Andrews NW. Plasma membrane repair is mediated by Ca(2+)-regulated exocytosis of lysosomes. Cell. 2001; 106: 157–169. 11511344 10.1016/s0092-8674(01)00421-4

[17] Gillies AR, Lieber RL. Structure and function of the skeletal muscle extracellular matrix. Muscle Nerve. 2011; 44: 318–331. 10.1002/mus.22094 21949456 PMC3177172

[18] Amaro A, Esposito AI, Gallina A, Nees M, Angelini G, Albini A, et al Validation of proposed prostate cancer biomarkers with gene expression data: a long road to travel. Cancer Metastasis Rev. 2014; 33: 657–671. 10.1007/s10555-013-9470-4 24477410 PMC4113682

[19] Kumar V, Vasudevan A, Soh LJ, Le Min C, Vyas A, Zewail-Foote M, et al Sexual attractiveness in male rats is associated with greater concentration of major urinary proteins. Biol Reprod. 2014; 91: 150 10.1095/biolreprod.114.117903 25359898

[20] Hui X, Zhu W, Wang Y, Lam KS, Zhang J, Wu D, et al Major urinary protein-1 increases energy expenditure and improves glucose intolerance through enhancing mitochondrial function in skeletal muscle of diabetic mice. J Biol Chem. 2009; 284: 14050–14057. 10.1074/jbc.M109.001107 19336396 PMC2682853

[21] O'Regan A, Berman JS. Osteopontin: a key cytokine in cell-mediated and granulomatous inflammation. Int J Exp Pathol. 2000; 81: 373–390. 11298186 10.1046/j.1365-2613.2000.00163.x PMC2517746

[22] Berman JS, Serlin D, Li X, Whitley G, Hayes J, Rishikof DC, et al Altered bleomycin-induced lung fibrosis in osteopontin-deficient mice. Am J Physiol Lung Cell Mole Physiol. 2004; 286(6): L1311–8. 10.1152/ajplung.00394.2003 14977630

[23] Denhardt DT, Noda M, O'Regan AW, Pavlin D, Berman JS. Osteopontin as a means to cope with environmental insults: regulation of inflammation, tissue remodeling, and cell survival. J Clin Invest. 2001; 107(9): 1055–1061. 11342566 10.1172/JCI12980 PMC209291

[24] Duvall CL, Taylor WR, Weiss D, Wojtowicz AM, Guldberg RE. Impaired angiogenesis, early callus formation, and late stage remodeling in fracture healing of osteopontin-deficient mice. J Bone Miner Res. 2007; 22: 286–297. 17087627 10.1359/jbmr.061103

[25] Giachelli CM, Lombardi D, Johnson RJ, Murry CE, Almeida M. Evidence for a role of osteopontin in macrophage infiltration in response to pathological stimuli in vivo. Am J Pathol. 1998; 152(2): 353–358. 9466560 PMC1857977

[26] Hashimoto M, Sun D, Rittling SR, Denhardt DT, Young W. Osteopontin-deficient mice exhibit less inflammation, greater tissue damage, and impaired locomotor recovery from spinal cord injury compared with wild-type controls. J Neurosci. 2007; 27: 3603–3611. 17392476 10.1523/JNEUROSCI.4805-06.2007 PMC6672107

[27] Liaw L, Almeida M, Hart CE, Schwartz SM, Giachelli CM. Osteopontin promotes vascular cell adhesion and spreading and is chemotactic for smooth muscle cells in vitro. Circ Res. 1994; 74: 214–224. 8293561 10.1161/01.res.74.2.214

[28] Mori R, Shaw TJ, Martin P. Molecular mechanisms linking wound inflammation and fibrosis: knockdown of osteopontin leads to rapid repair and reduced scarring. J Exp Med. 2008; 205: 43–51. 10.1084/jem.20071412 18180311 PMC2234383

[29] O’Regan A, Berman JS. Osteopontin: a key cytokine in cell-mediated and granulomatous inflammation. Int J Exp Pathol. 2000; 81: 373–390. 11298186 10.1046/j.1365-2613.2000.00163.x PMC2517746

[30] Sam F, Xie Z, Ooi H, Kerstetter DL, Colucci WS, Singh M, et al Mice lacking osteopontin exhibit increased left ventricular dilation and reduced fibrosis after aldosterone infusion. Am J Hypertens. 2004; 17: 188–193. 14751663 10.1016/j.amjhyper.2003.10.007

[31] Haslett JN, Sanoudou D, Kho AT, Bennett RR, Greenberg SA, Kohane IS, et al Gene expression comparison of biopsies from Duchenne muscular dystrophy (DMD) and normal skeletal muscle. Proc Natl Acad Sci USA. 2002; 99: 15000–15005. 12415109 10.1073/pnas.192571199 PMC137534

[32] Hoffman EP, Gordish-Dressman H, McLane VD, Devaney JM, Thompson PD, Visich P, et al Alterations in osteopontin modify muscle size in females in both humans and mice. Med Sci Sports Exerc. 2013; 45: 1060–1068. 10.1249/MSS.0b013e31828093c1 23274598 PMC3631433

[33] Pagel CN, Wijesinghe DKW, Esfandouni NT, Mackie EJ. Osteopontin, inflammation and myogenesis: influencing regeneration, fibrosis and size of skeletal muscle. J Cell Commun Signal. 2014; 8(2): 95–103. 10.1007/s12079-013-0217-3 24318932 PMC4063988

[34] Uaesoontrachoon K, Yoo HJ, Tudor EM, Pike RN, Mackie EJ, Pagel CN. Osteopontin and skeletal muscle myoblasts: association with muscle regeneration and regulation of myoblast function in vitro. Int J Biochem Cell Biol. 2008; 40: 2303–2314. 10.1016/j.biocel.2008.03.020 18490187

[35] Coley WD, Bogdanik L, Vila MC, Yu Q, Van Der Meulen JH, Rayavarapu S, et al Effect of genetic background on the dystrophic phenotype in mdx mice. Hum Mol Genet. 2016; 25(1): 130–145. 10.1093/hmg/ddv460 26566673 PMC4690497

[36] Fukada S, Morikawa D, Yamamoto Y, Yoshida T, Sumie N, Yamaguchi M, et al Genetic background affects properties of satellite cells and mdx phenotypes. Am J Pathol. 2010; 176(5): 2414–2424. 10.2353/ajpath.2010.090887 20304955 PMC2861106

[37] Fatherazi S, Matsa-Dunn D, Foster BL, Rutherford RB, Somerman MJ, Preland RB. Phosphate regulates osteopontin Gene transcription. J Dent Res. 2009; 88(1): 39–44. 10.1177/0022034508328072 19131315 PMC3128439

[38] Subraman V, Thiyagarajan M, Malathi N, Rajan ST. OPN–Revisited. J Clin Diagn Res. 2015; 9(6): ZE10–ZE13. 10.7860/JCDR/2015/12872.6111 26266236 PMC4525627

[39] Agnihotri R, Crawford HC, Haro H, Matrisian LM, Havrda MC, Liaw L. Osteopontin, a novel substrate for matrix metalloproteinase-3 (stromelysin-1) and matrix metalloproteinase-7 (matrilysin). J Biol Chem. 2001; 276: 28261–28267. 11375993 10.1074/jbc.M103608200

[40] Takafuji V, Forgues M, Unsworth E, Goldsmith P, Wang XW. An osteopontin fragment is essential for tumor cell invasion in hepatocellular carcinoma. Oncogene. 2007; 26(44): 6361–6371. 17452979 10.1038/sj.onc.1210463

[41] Lund AK, Lucero J, Lucas S, Madden MC, McDonald JD, Seagrave JC, et al Vehicular emissions induce vascular MMP-9 expression and activity associated with endothelin-1-mediated pathways. Arterioscler Thromb Vasc Biol. 2009; 29(4): 511–517. 10.1161/ATVBAHA.108.176107 19150882 PMC4103743

[42] Vetrone SA, Montecino-Rodriguez E, Kudryashova E, Kramerova I, Hoffman EP, Liu SD, et al Osteopontin promotes fibrosis in dystrophic mouse muscle by modulating immune cell subsets and intramuscular TGF-beta. J Clin Invest. 2009; 119: 1583–1594. 10.1172/JCI37662 19451692 PMC2689112

[43] Pegoraro E, Hoffman EP, Piva L, Gavassini BF, Cagnin S, Ermani M, et al Cooperative International Neuromuscular Research. SPP1 genotype is a determinant of disease severity in Duchenne muscular dystrophy. Neurology. 2011; 76: 219–226. 21178099 10.1212/WNL.0b013e318207afeb PMC3034396

